# The Amaurosis and Deafness of Smokers and Drinkers

**Published:** 1866-03

**Authors:** 


### THE AMAUROSIS AND DEAFNESS OF SMOKERS AND DRINKERS.

By MM. SICHEL AND TRIQUET.

*Annales d'Oculistique*, Mars; and *Gazette des Hopiteaux.*

M. Sichel, in continuation of a former communication published in 1863, observes that among the forms of cerebral amaurosis there are two which, although little known, are not of infrequent occurrence, and are difficult of cure. One of these, produced by the abuse of alcoholic drinks, he described as long ago as 1837, under the designation of "amaurosis symptomatic of delirium tremens;" and the other, produced by the abuse of smoking, was first described by Mackenzie. Incredulous, as to this last, when first announced, M. Sichel, in the course of 28 years' practice, has frequently met with it, and he believes that there are few persons who can smoke for any long period more than five drachms of tobacco daily, without their vision, and often their memory, becoming affected. In both these forms of amaurosis there is well-nigh absence of all well-marked cerebral congestion, and there is a vagueness in their sthenic or asthenic characters, which may cause hesitation and perplexity on the part of the surgeon, if unaware of the cause in operation. The ophthalmoscopic appearances, as in most old cerebral amauroses, are negative or ill-marked. The optic papillæ, sometimes very white, especially in one of their halves, sometimes slightly injected, have their contours ill-circumscribed or in part effaced. The retina is but little injected, the central vessels being sometimes normal and sometimes enlarged, the central veins being especially so when the affection has reached its last stage. All the characters observed are, in fact, in common with those of other cerebral amauroses. As in many of these, too, the memory is often enfeebled; and in the amaurosis from alcohol there are frequently trembling of the hands in the morning, and at a later period morning vomiting. Both of these varieties are very slow in their progress toward cure, and very refractory to treatment. Usually observed separately, they may be seen together, and in such cases it is not easy to decide whether the tobacco or the alcohol plays the chief part. The treatment of these cases usually occupies a long time, and an essential point, of course, is the discontinuance of the practice that has given rise to the amblyopia or amaurosis. In the few cases in which there is any marked congestion present, this must be met by antiphlogistics; but when this is not very positive, bleeding must only be resorted to with the greatest care. As in all forms of passive or old cerebro-ocular congestion, liberal depletion, even by leeching or cupping, and still more even moderate bleeding, soon completes the loss of vision, and this is only slowly and incompletely restored. On the other hand, external and internal stimulants, such as liniments, flying blisters, camphor, strychnine, etc., resorted to before a moderate antiphlogistic and derivative treatment has been put into force, only aggravate the disease. When there is but little congestion, mild aperients are very useful, such as equal parts of cream of tartar and magnesia, alternating with pills of gum ammoniac, sulphate of potass, and aloes. In drinkers these means will not be borne, and minute doses of rhubarb and magnesia may be substituted. Cold water should be applied to the forehead and eyes, while the lower extremities are irritated by sinapisms, dry cupping, etc. At a later period are indicated stimulant liniments to the circumorbital region, flying blisters, first to the nape, or behind the ears, and then to the temples; and, in very obstinate cases, the various internal stimuli, as camphor, arnica, strychnine, etc., may be tried.

M. Triquet states that in smokers and drinkers an insidious and obstinate form of otitis frequently becomes developed. There is a kind of numbness or torpor of the ear, with a sense of cold, but rarely any pain. There is no cerumen in the meatus, the membrane and ossicula are in a normal state, and there is little or no vascularity. There is, however, extreme dryness with very minute granulations of the pharynx, nasal fossæ, tubes, and middle ear. Frequently both ears are affected, but one has always commenced being so before, and is more deaf than the other. The deafness, without being very troublesome at first, rapidly increases. Noises in the ear almost always exist at an early period, and it is of importance to notice that they assume a hissing sound. The affection exhibits itself in three periods:—1, that of excitement, in which there is intolerance of noise, and a hissing noise in the ear; 2, that of depression, in which the hissing sound disappears, or only remains as a distant and feeble echo; and 3, that of a paralytic condition of the auditory nerve, in which the sense of hearing is more or less completely, and often permanently, lost. In this period there are also often trembling of the tongue, embarassment of speech, and disturbance of vision. The prognosis is very unfavorable, for those patients alone are susceptible of cure who will consent to leave off the bad habit which has produced the affection. For treatment, in the very early stages, cupping of the mastoid processes and drastic purgatives, and then alteratives, as calomel, sulphur, and small doses of arsenic, are indicated. Locally stimulating fumigations, and weak injections of strychnine or veratrine have proved useful; electricity has always done harm.—*British & For. Med.-Chir. Review, and Dental Cosmos.*

